# Effect of topographical control by a micro-molding process on the activity of human Mesenchymal Stem Cells on alumina ceramics

**DOI:** 10.1186/s40824-015-0045-z

**Published:** 2015-11-04

**Authors:** Soo-Yean Kim, Jong-Ho Kang, Won-Seon Seo, Suk-Won Lee, Nam-Sik Oh, Hyung-Koun Cho, Myung-Hyun Lee

**Affiliations:** Energy & Environmental Division, Korea Institute of Ceramic Engineering & Technology, 101, Soho-ro, Jinju-si, Gyeongsangnam-do 660-031 Republic of Korea; Department of Biomaterials & Prosthodontics, Kyung Hee University Hospital at Gangdong, Institute of Oral Biology, School of Dentistry, Kyung Hee University, 892 Dongnam-ro, Gangdong-gu, Seoul 134-727 Republic of Korea; Department of Dentistry, College of Medicine, Inha University, 27 Inhang-ro, Jung-gu, Incheon 400-711 Republic of Korea; Department of Advanced Materials Science & Engineering, Sungkyunkwan University, Suwon, 440-746 South Korea

**Keywords:** Microgroove, Alumina, Human Mesenchymal Stem Cell, Micro-molding, Polydimethylsiloxane

## Abstract

**Background:**

Numerous studies have reported that microgrooves on metal and polymer materials can affect cell adhesion, proliferation, differentiation and guidance. However, our knowledge of the cell activity associated with microgrooves on ceramics, such as alumina, zirconia, hydroxyapatite and etc, is very incomplete, owing to difficulties in the engraving of microgrooves on the hard surface of the base material. In this study, microgrooves on alumina were fabricated by a casting process using a polydimethylsiloxane micro-mold. The cell responses of Human Mesenchymal Stem Cells on the alumina microgrooves were then evaluated.

**Results:**

Microgrooves on an alumina surface by micro-mold casting can enhance the adhesion, differentiation of osteoblasts as well as gene expression related to osteoblast differentiation. The ALP activity and calcium concentration of the cells on alumina microgrooves were increased by more than twice compared to a non-microgrooved alumina surface. Moreover, regarding the osteoblast differentiation of hMSCs, the expression of ALP, RUNX2, OSX, OC and OPN on the microgrooved alumina were all significantly increased by 1.5 ~ 2.5 fold compared with the non-microgrooved alumina.

**Conclusion:**

Altering the topography on alumina by creating microgrooves using a micro-molding process has an important impact on the behavior of hMSCs, including the adhesion, differentiation of osteoblasts and osteoblast-specific gene expression. The significant increase in hMSC activity is explained by the increasing of material transportation in parallel direction and by the extending of spreading distance in perpendicular direction.

## Background

Microgrooves have been reported to be effective for altering cell shape, changing cell adhesion and proliferation on polystyrene [1], titanium [2] and silicon [3]. Alumina is a bio-inert ceramic that is used in dental and orthopedic applications due to its chemical stability, mechanical properties and biocompatibility [4]. However, applying micropatterns to alumina by using conventional microfabrication techniques such as micromachining, laser cutting and etching techniques [5] is very difficult, due to its brittleness, hardness and inertness [6, 7]. Danish Nadeem et al., reported on the fabrication of Mesenchymal Stem Cells (MSCs) on alumina micropatterns with widths of 50 μm and cell alignment on 10 μm widths using an embossing technique [8]. A few related reports have reviewed cell guidance and cell differentiation on microgrooved alumina. In addition, information concerning hMSC behavior, including gene expression related to osteoblastic differentiation on microgrooved alumina in a wide range to 180 μm, which approaches the width of microgrooves for a micromachined titanium implant, is insufficient.

In this study, microgrooved alumina was fabricated using a micro-molding process. Such a process involved fabricating a microgrooved alumina substrate by casting slurry on a polydimethylsiloxane (PDMS) replica, drying to a green body and subsequently sintering to produce a dense body. The procedure is capable of transferring geometries and surface details of a titanium mother model which was micropatterned by photolithography [9]. The activity of human Mesenchymal Stem Cells (hMSCs) as well as gene expression on the microgrooved surfaces of alumina was investigated. Furthermore, we compared the surface chemistry, surface hydrophilicity, cell adhesion, activity and maturation on titanium with those on alumina. The effect of surface chemistry and material properties on cell response was also evaluated.

## Methods

### Fabrication of microgrooved surfaces on alumina ceramic

**Fig. 1:**
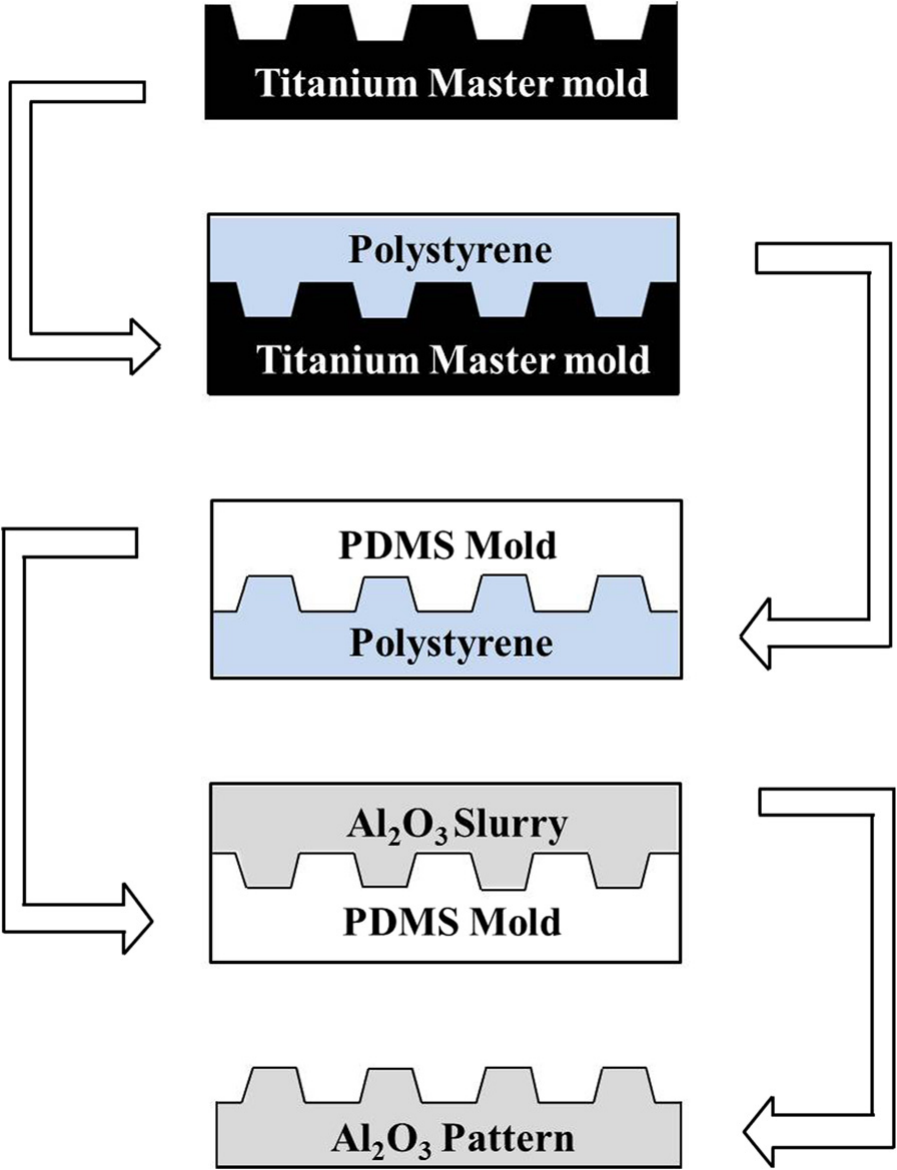
A schematic drawing of the experimental procedure for the microgrooved alumina

**Fig. 2:**
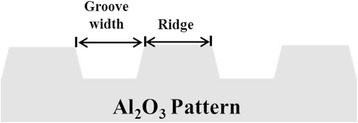
Schematic cross-sectional image and the structural nomenclature of the fabricated microgrooved alumina surface

The replicating and molding process for preparing microgrooved patterns on alumina ceramics are shown schematically in Fig. 1. The titanium mother substrates used in the study were prepared with the commercially pure titanium foil with a thickness of 0.14 mm (CP-Ti, ASTM Grade II, TSM-Tech Co. Ltd., Korea). The titanium surface was buffed with emery powder compounds and ground using a cloth wheel. Polished titanium was cleaned using isopropyl alcohol (IPA), acetone and distilled water in an ultrasonicator. The photolithography process was performed for a microgrooved titanium mother model [2] for various pattern widths in the range of 30 ~ 180 μm. The titanium mother model was replicated onto a polystyrene (PS) substrate by pressing unidirectionally with a pressure of 1000 Kgf/cm^2^ at 120 °C for 20 s. After replicating the micropattern of titanium mother substrate on the PS substrate, an acrylic tube was attached to it for PDMS processing. PDMS solution was poured onto the acrylic tube on PS replica substrate and kept at 120 °C for curing. After curing and cooling, microgrooved PDMS replica disc was surrounded with a polyethylene film to a height of 5 mm to maintain the alumina slurry during the drying process. Alumina powder **(**AES-11, Sumitomo Chemical Co., Japan**)** with particle sizes of approximately 0.5 μm, PVA (Poly vinyl alcohol, Mw 85000–124000, Sigma Aldrich, USA), dispersant (Darvan C, Vandervilt Co. Inc., USA) and antifoamer (NOPCO NXZ, San Nopco Korea Ltd., Korea) were mixed in D.I water and ball-milled in a polyethylene jar using zirconia media (diameter of 5, 10 mm) for 24 h. After ball-milling, a primary defoamation process was performed at 1.3 × 10^−1^Pa using a vacuum pump (G-50DA, ULVAC, Japan). The alumina slurry was then cast onto the PDMS replica mold. A second defoamation process was carried out to remove pores that were trapped between the slurry and the surface of the replica mold during casting. After the casting and defoamation process, the alumina slurry was dried. The drying process was carried out in a temperature/humidity chamber (SH-241, ESPEC CORP, Japan) maintained at a temperature of 40 °C with a relative humidity of 80 % for 25 h. The dried specimen was then detached from the PDMS replica mold. The alumina specimen was sintered at 1650 °C for 1 h in air. A control specimen (NE0) and mother substrates were buffed and ground following the same process was used in the preparation method for photolithography [7]. A schematic cross-sectional image and the structural nomenclature of the fabricated microgroove alumina surface prepared using micromolding process is shown in Fig. 2. The specimen groups used in this study with ridges and grooves of varying widths are listed in Table 1.

**Table 1:** Titanium and alumina substrate with various surface topographies used in this study (μm)

Specimen	Ridge width [μm]	Groove width [μm]
NE0	0	0
AM0	0	0
AM30	30	30
AM60	60	60
AM90	90	90
AM120	120	120
AM150	150	150
AM180	180	180

### Surface characterization

The surface morphology and roughness ratio (r; relative value of measured surface area to unit area) of specimens was characterized by a confocal laser scanning microscopy (OLS3000-300 mm autostage, Olympus, Japan) with a 408-nm argon laser excitation source. The contrast in the confocal laser-scanning microscope is based on the reflection of the laser beam from the specimen surface. Thus, point-by-point images were reconstructed under small point scans in the X-Y direction. The percentage of area in the spectra of C1s and O1s bonding on the titanium and the alumina surface was characterized using X-ray photoelectron spectroscopy (XPS, PHI5000, Physical Electronics, U. S. A). XPS experiments were performed in an ultra-high vacuum using Al Ka (hv = 1486.6 eV) radiation. High resolution XPS analysis of the C1s and O1s peaks was performed in the range of 280–295 eV and 526–536 eV with a step of 0.05 eV, respectively. The scan spectra were analyzed by skewed Gauss-Lorenz Line shapes using the XPS peak 4.0 software associated with the XPS instrumentation. A drop shape analysis system (EasyDrop standard, KRUSS GmbH, Germany) was used for the water contact angle (WCA) measurements. A 6 μl drop of distilled water was used as a probe, and measurements were taken for each drop. The drop images were captured serially five times every 5 s by a video camera. The water contact angle of each surface was calculated using an image analysis software program. WCA analyses are performed separate in parallel and perpendicular directions to the microgrooves.

### Cell culture

Human bone marrow-derived mesenchymal stem cells (hMSC) were purchased from Lonza (Lonza, MD, USA) and grown in MSC Growth Medium (MSCGM; Lonza, MD, USA) at 37 °C with 5 % CO_2_ and 95 % humidity. Tissues were obtained from subjects following informed consent guidelines as described by the Institutional Review Board protocol of Kyung Hee University Hospital at Gangdong. Cells were maintained in Dulbecco’s Modified Eagle’s Medium (DMEM: Welgene, Daegu, Korea) containing 10 % FBS (fetal bovine serum, Sigma-Aldrich, St. Louis, MO, USA), 100U/ml penicillin, and 100 μg/ml streptomycin at 37 °C under 5 % CO_2_. MSCs at passages 3–5 were used in this study.

### Cell Count Kit (CCK) cell adhesion assay

MSCs were seeded on the 24-well titanium and alumina surfaces at a concentration of 1 × 10^4^ cells/well and cultured for 16 h at 37 °C under 5 % CO_2_. Twenty microliters of Cell Counting Kit reagent (50 ml, CCK-8; Dojindo, Kumamoto, Japan) were added to each well followed by incubation for 2 h. The reaction products were transferred to 96-well plates and monitored using a microplate reader (Bio-Rad, Hercules, CA, USA) at 450 nm.

### Alkaline phosphatase activity assay

MSCs were seeded on the 24-well titanium and alumina surface at 4 × 10^4^ cells/well and cultured for 2 days to achieve confluence. The cells were then cultured in osteogenic media [DMEM supplemented with 10 % FBS (Invitrogen), 50 μg/ml α-ascorbic acid (Sigma-Aldrich, St. Louis, MO, USA), 10 mM β-glycerophosphate (Sigma-Aldrich), 100 mM dexametasone (Sigma-Aldrich)] at 37 °C under 5 % CO_2_ for 14 days to investigate ALP activity. The reaction products were transferred to 96-well plates and monitored using a microplate reader (Bio-Rad, Hercules, CA, USA) at 405 nm, and measurements were compared using pnitrophenol standards and normalized to total protein levels.

### Extracellular calcium deposition assay

Extracellular calcium deposition on the titanium and alumina surface was quantified to investigate the steps of osteoblast differentiation. MSCs was seeded on 24-well titanium and alumina surfaces at a concentration of 4 × 10^4^ cells/well and cultured for 2 days to achieve confluence. Cells were then cultured in osteogenic media at 37 °C under 5 % CO_2_ for 21 days. Cells were rinsed in a phosphate buffered solution (PBS, Gibco BRL, Grand Island, NY, USA) and the surface with remaining calcium deposits were incubated with 0.5 N HCl at 4.8 °C overnight. Following centrifugation, the amount of calcium present in the acidic supernatant was quantified using Calcium Liquicolor (Stanbio Laboratory, Boerne, TX, USA). Light absorbance was measured using a microplatereader (Bio-Rad) at 650 nm. Total calcium (μg/well) was calculated from standard curves derived from absorbance values versus the calcium levels of controls measured in parallel with the experimental surfaces.

### Osteoblast-related gene expression

The relative mRNA expression of five osteo-related genes was analyzed in the hMSCs using quantitative real-time PCR. The five genes analysed were RUNX2 (run-related trascription factor2), OSX (osterix), ALP (alkaline phosphatase), OCN (osteoclacin), OPN (osteopontin). MSCs was seeded on 24-well alumina surface at a density of 4 × 10^4^ cells/well and incubated for 2 days until reaching confluence. Cells were then cultured for 14 days in osteogenic media at 37 °C under 5 % CO_2_. Total RNA was extracted using Trizol (Invitrogen). RNA concentration was determined using a NanoDrop 1000 (Nano-Drop Technologies, Wilmington, DE, USA). An iScript cDNA Synthesis Kit (Bio-Rad) with 1 mg of total RNA was used to reverse-transcribe cDNA. The mRNA expression levels of the five osteo-related genes were determined relative to the internal GAPDH control using a TaqMan1 Gene Expression Assay Kit (Applied Biosystems, Foster City, CA, USA). Chromo4 Reverse Transcription-Polymerase Chain Reactions (Bio-Rad Laboratories, Hemel Hempstead, UK) were performed using IQ Supermix (Bio-Rad). The MJ Opticon Monitor Analysis Software (Bio-Rad) was used to quantify gene expression levels. Experimental values were normalized to GAPDH in order to obtain relative expression levels.

### Statistical analyses

The CCK cell adhesion, ALP activity test, extracellular calcium deposition assays, and quantitative real-time PCR of hMSCs on titanium and alumina surface were performed simultaneously and independently four times, and the mean values from these experiments were compared using a one-way analysis of variance (ANOVA). SPSS 17.0 software was used for all statistical analyses.

## Results & discussion

### Surface chemistry and morphology of micro-patterned surfaces

**Fig. 3:**
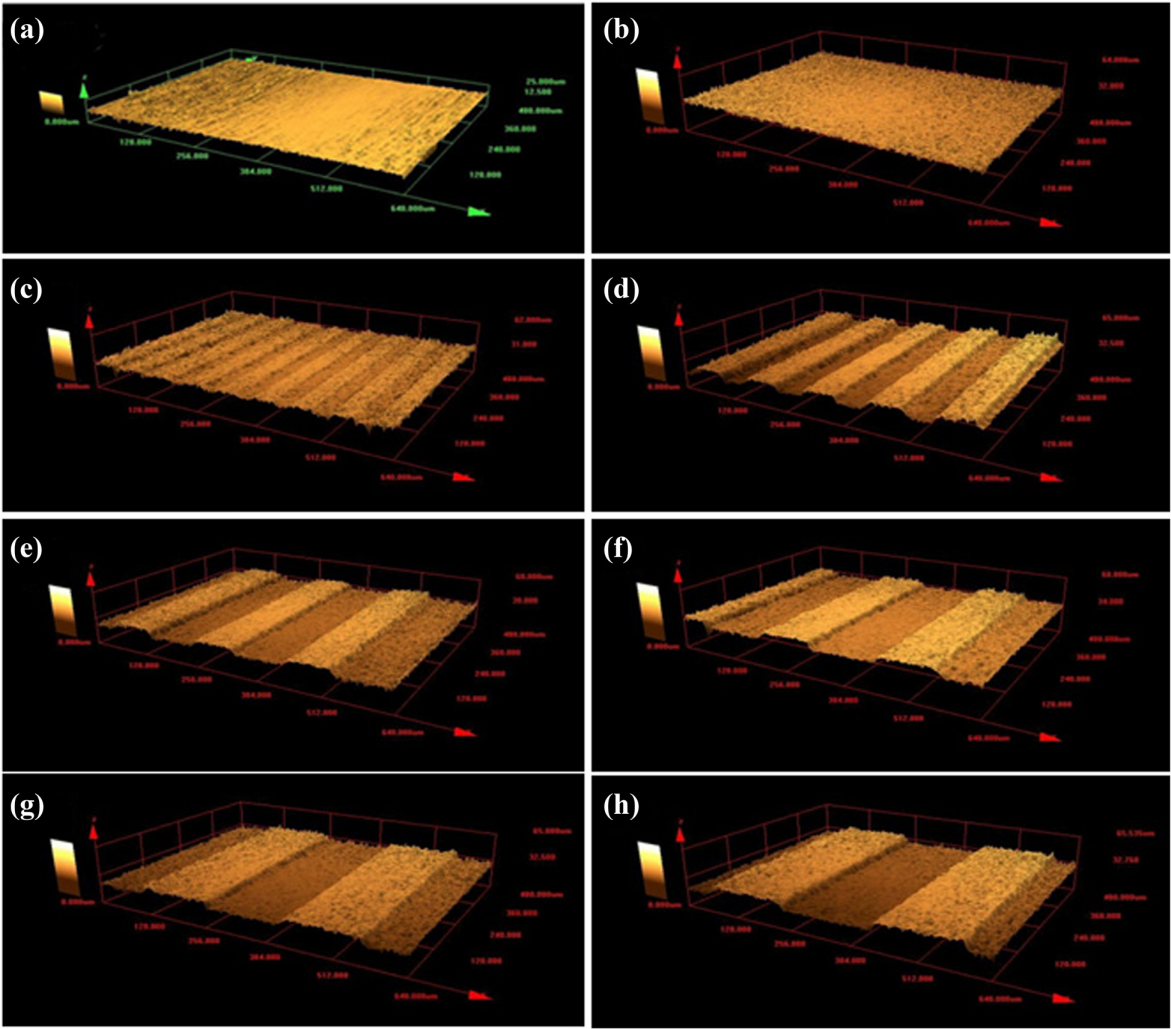
Confocal Laser Scanning Microscopy (CLSM) images of the fabricated surfaces of NE0 and samples patterned with the width of from 30 to 180 μm, **a** NE0, **b** AM0, **c** AM30, **d** AM60, **e** AM90, **f** AM120, **g** AM150 and **h** AM180

The dimension and uniformity of microgrooves and ridges in the patterned alumina substrates prepared by a micro-molding technique were examined by confocal laser scanning microscopy (CLSM) (Fig. 3). The finding confirmed that the pattern of the titanium substrate was effectively transferred to the alumina substrate using this process. Each specimen with designed groove widths of 30, 60, 90, 120, 150 and 180 μm showed an error range of 5 % from the original design after a 3-step replicating of molding and sintering of the cast alumina. The average roughness of the un-patterned titanium (NE0) and alumina (AM0) surface was 0.15 ± 0.02 μm and 0.72 ± 0.06 μm, respectively (data not shown). The effects of surface microgrooves in various materials on osteoblast activities, such as polystyrene [1] titanium [2] and silicon [3] have been examined in previous studies. Only a very few studies dealing with the surfaces of microgrooved ceramics such as alumina, titania and zirconia have been reported. Conventional microfabrication techniques such as photolithography cannot achieve a satisfactory target shape and accuracy because of the high chemical stability of ceramics [7]. CNC (Computer Numerical Control) machining or laser machining has also been reported, but the disadvantages of these techniques include low accuracy and the high cost of equipment [8, 9]. Therefore, additive approaches such as micro-transfer molding and direct jet printing have been used [10], where a ceramic suspension is deposited on the surface of a material with the same or a different composition. However, these techniques have disadvantages, such as a low interfacial bonding strength between the substrate and jetted ceramic after sintering. For the micropatterning of ceramics, applying micro-molding techniques using a soft mask or mold such as PDMS can be used efficiently without the need for an etching or machining process. Also, it is possible to fabricate ceramic bodies with a micropatterned surface with various geometries on the surface [11].

**Fig. 4:**
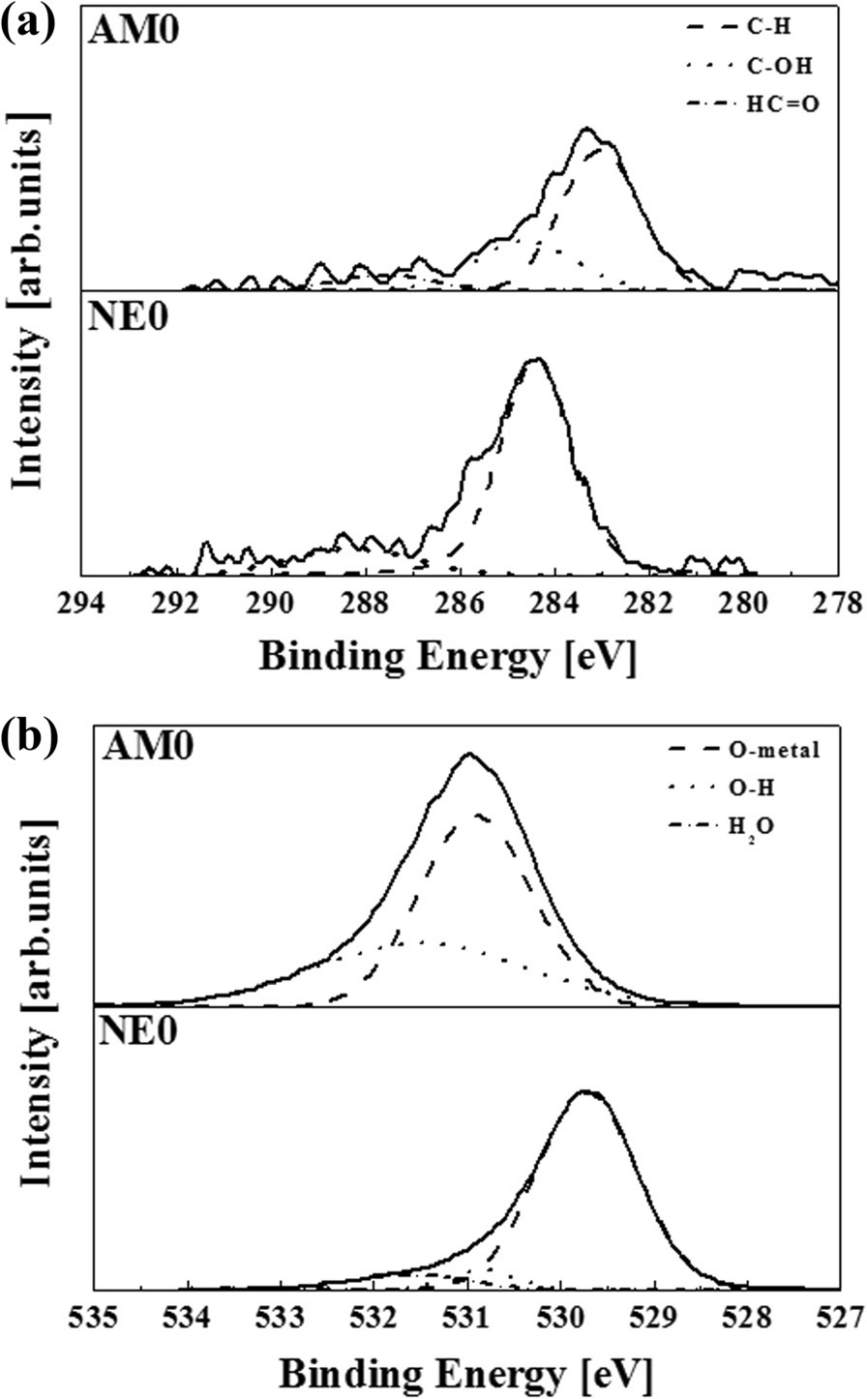
High-resolution XPS spectra of **a** C1s and **b** O1s of the titanium and alumina surface

**Fig. 5:**
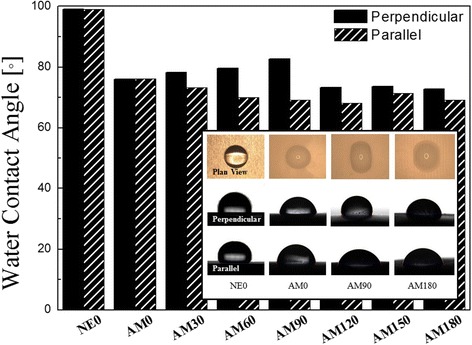
The water contact angles (WCAs) and images of droplet measured in the directions parallel with and perpendicular to the microgroove

The XPS analysis confirmed the chemical components and bonding on each surface. XPS spectra of C1s and O1s core levels are presented in Fig. 4. The C1s spectra of the NE0 surface can be resolved into three peaks at 284.35, 285.79 and 288.24 eV which can be attributed to the binding energy of C-H, C-OH and HC = O group, respectively. The C1s spectra of AM0 surface can also be resolved into three peaks at 284.09, 285.39 and 287.79 eV, which can be assigned to the binding energy of C-H, C-OH and HC = O groups, respectively. The area percentage of C-H bonding group on the NE0 and AM0 surface was 74.2 and 63.2 %, respectively. The amount of C-H groups on the NE0 surface was higher than that on the AM0 surface. O1s spectra of the NE0 surface can be resolved into two peaks at 529.77 and 531.67 eV binding energy which can be attributed to Ti-O and O-H groups, respectively. O1s spectra of the alumina surface can be resolved into two binding energy peaks at 530.86 and 531.45 eV that are assigned to Al-O and O-H groups, respectively. The area percentages of the C-OH bonding group on the titanium and alumina surface were 10.24 and 27.7 %, respectively. In addition, the area percentages of O-H bonding group on the NE0 and AM0 were 13.71 and 39.78 %, respectively. An alumina surface has a significantly high area percentage of both O-H and C-OH bonding compared with a titanium (NE0) surface. The surface characteristics of biomaterials including chemical components and charge state can affect cell responses to implants. The results of this study indicate that, cell adhesion, ALP activity and calcium deposition are significantly higher on alumina surfaces than on titanium surfaces. The XPS result for the O1s core showed that the concentration of O-H bonds on the alumina surfaces is higher than that on titanium surfaces. According to Tari et al., the adsorptive behavior of hydroxyl groups on alumina can be attributed to aluminum atoms at the surface, which are electron deficient and form hydrogen bonds with interfacial water molecules that are present in the atmosphere [12]. Hydrophilicity can be assessed by measuring the contact angle through the spread of a water droplet on the surface of a material (Fig. 5). The hydrophilicity increased with decreasing as water contact angle. The WCA of the AM0 surface, 76.1°, was lower than that of the NE0 surface, 89°. In these results, the AM0 surface was found to be relatively hydrophilic compared with the NE0 surface. The difference in surface chemical composition between alumina and titanium influenced the alteration of WCA. It is known that hydroxyl groups at material surfaces can cause a surface to be more hydrophilic [13] but hydrocarbon groups, to be more hydrophobic. [14]. Therefore, an alumina surface is more hydrophilic than a titanium surface. It is well known that surface hydrophilicity is a key factor in cell growth. Many papers have suggested that a greater concentration of surface hydroxyl groups resulted in greater numbers of attached osteoblasts and a higher cell activity [15, 16] Therefore, we suggest that a high concentration of hydroxyl groups and low levels of hydrocarbon groups on the alumina surface results in a material with a high cell activity compared with a titanium surface. In addition, surface charges affect cell attachment and growth [17]. The attachment and spreading of cells are influenced by the surface charge, i.e., whether the surface is positively or negatively charged [18]. The attachment and spreading of osteoblasts is more localized on positively charged regions than on negatively charged surfaces [19]. As a quantitative measure the isoelectric point (IEP) is often used to evaluate surface charge. In an evaluation of the IEP for alumina and titania surfaces, the values for alumina and titania were determined to be 8.8–9.5 and 3.5–6.7, respectively [18]. The surface tends more positive when the IEP exceeds pH 7.4 [18]. Therefore, an alumina surface has a more positive charge than titanium which contains a native oxide layer of titania on its surface. Also, in our study, the amount of hydroxyl groups on the alumina surface was higher than that on titanium. It is thought that the positive charge and hydroxyl groups of an alumina surface affect the attachment and spreading of osteoblasts. Consequently, the chemical structure and charges of a material surface plays an important role in enhancing cell activity.

WCA could be affected by not only surface chemistry but also by surface topography. WCA analyses were performed separately in both parallel and perpendicular directions to the microgrooves. Since the surface chemistry was the same in all samples except titanium (NE0), the difference in WCA can be assumed to be exclusively due to the presence of surface microgrooves.

The WCA in the perpendicular direction to the microgrooves was increased from 76.1° for AM0 to 82.6° for AM90 and decreased to 73° for AM120, AM150 and AM180. Increasing of WCA in the perpendicular direction by increasing up to AM90 could be explained by the Wenzel equation [20], which is the relationship between the contact angle and surface roughness.

1 $$ \cos \uptheta * = \mathrm{r}\kern0.5em  \cos \uptheta $$

where r: roughness ratio, θ: wetting contact angle, θ*: apparent contact angle on rough surface. By increasing the width of microgroove from 0 to 90 μm, roughness ratio (r) of CLSM analyses was increased from 1.0 to 1.035. In the microgroove of the width of 120 μm, it was decreased and kept to around 1.020 in the wider microgrooves. By following the Wenzel equation, the apparent angle on a rough surface (θ*) increases with increasing roughness.

In the observation of WCA in the perpendicular direction to the microgroove, it was anticipated that capillary pressure would be higher as the width became narrower and that this could enhance the anisotropy of a water droplet on a microgroove substrate. However, anisotropy was increased with increasing width of the microgroove in these analyzed specimen groups and was highest for the AM90 sample. Therefore the wetting angle of a water drop was evaluated as the highest in the perpendicular direction on the AM90 sample. Up to a microgroove width of 90 μm, it was observed that grooves worked as a breakwater to advance the water droplet in the perpendicular direction. Hence, we suggest that the anisotropy of the water droplet is due to, not only the penetration of water into the grooves via a capillary effect in the parallel direction [21] but also hindering the advancing of a water droplet in the perpendicular direction. The WCA of microgrooved alumina in the parallel direction was decreased with increasing width of the microgroove. In the parallel direction, it was observed that the water droplet spread over a longer distance, which would seem to be originated from improving wettability and transportation by the microgroove. In comparison with the spreading distance of water droplet between specimens, the differences are relatively small in the parallel direction than in the perpendicular direction. The wide microgroove acted as a transport channel for water and induced isotropic and long spreading of droplet in the tested specimen groups.

### In-vitro test

Many studies have been reported on cell–biomaterial interactions with various micropatterned surfaces [22, 23]. The presence of a micropattern on a surface can have significant effects on cellular behavior, including cell adhesion, migration, proliferation, and differentiation [24]. A large number of studies have been carried out in attempts to examine the effect of microgrooves on different types of cells. Other investigations reported that the attachment of fibroblasts is increased on smooth surfaces and soft tissue growth, whereas the attachment of osteoblasts is increased on rough surfaces and in bone growth [25].

**Fig. 6:**
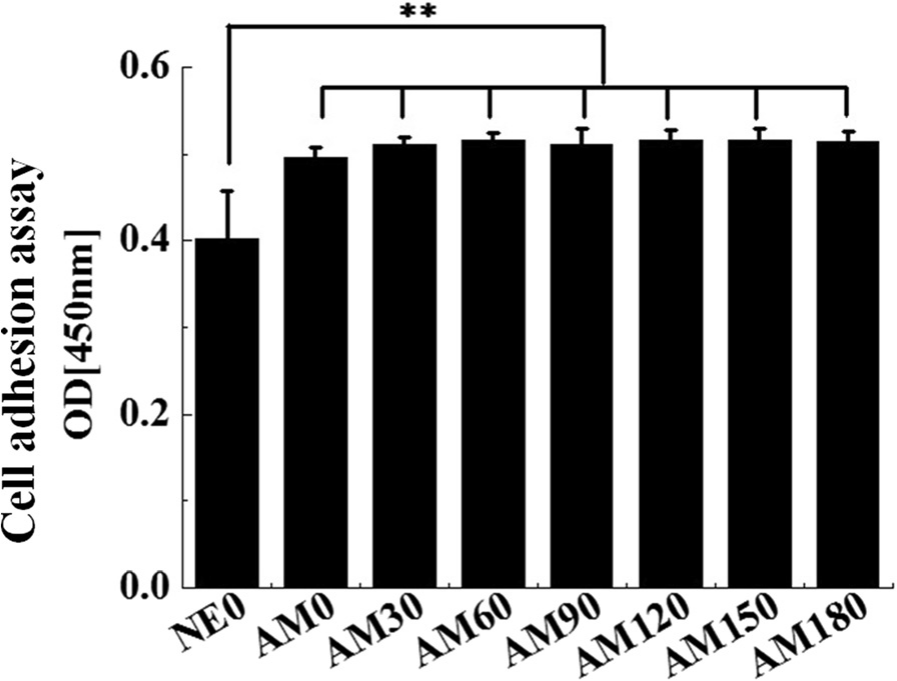
Multiple-comparison result of the cell adhesion assay using Cell Count Kit after 16 h of culture on NE0 and alumina surface with surface topographies. Oneway ANOVA (*n* = 5). **: significant difference (*p* < 0.005)

**Fig. 7:**
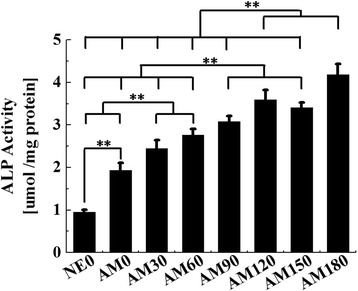
Multiple-comparison result of the alkaline phosphatase activity of human bone marrow-derived mesenchymal stem cells after 14 days of osteogenic culture on NE0 and alumina surface with surface topographies. Oneway ANOVA (*n* = 5). **: significant difference (*p* < 0.005)

**Fig. 8:**
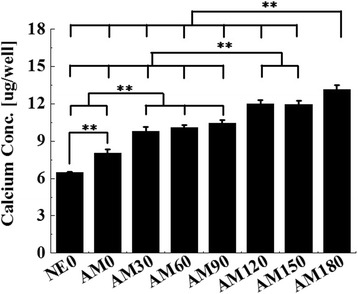
Multiple-comparison result of the osteoblast differentiation of human bone marrow-derived mesenchymal stem cells after 21 days of osteogenic culture on NE0 and alumina surface with various surface topographies using extracellular calcium deposition assay. Oneway ANOVA (*n* = 5). **: significant difference (*p* < 0.005)

**Fig. 9:**
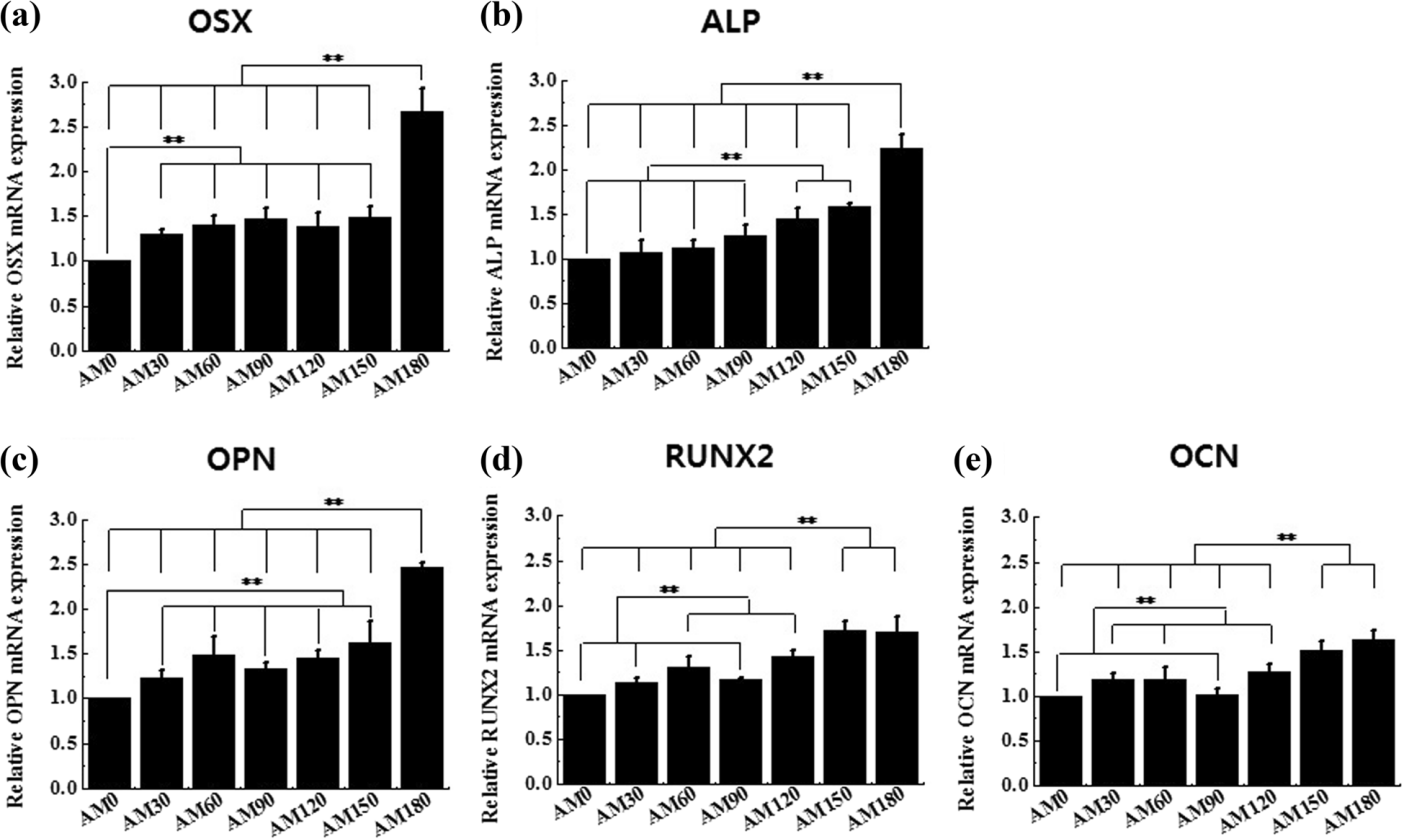
Relative fold change mRNA expression of **a** osterix (OSX), **b** alkaline phosphatase (ALP), **c** osteopontin (OPN), **d** runt-related transcription factor 2 (RUNX2), and **e** osteocalcin (OCN) in human bone marrow-derived mesenchymal stem cells after 14 days of osteogenic culture. Oneway ANOVA (*n* = 5). **: significant difference (*p* < 0.005)

We performed a cell adhesion assay using hMSCs cells that were incubated for up to 16 h in this study. We used a CCK-8 assay for the cell adhesion analyses and the results showed significantly enhanced differences between AM0 and patterned surfaces but no significant differences in the adhesion of hMSCs cells between the various patterned groups (Fig. 6). These results are in agreement with previous reports indicating that there are no significant differences in the attachment of osteoblasts between non-microgrooved and microgrooved titanium surfaces [26] but contradict another study that reported a significant increase in cell adhesion on microgrooves. It assumed that the surface topography had less effect in the initial in-vitro conditions because the cell adhesion assay was measured after incubating for a period of up to 16 h. Alkaline phosphatase is an important component of cells and is expressed at high levels in mineralized cells. ALP activity of the cells on the AM0 surface after a 14 day maturation period was significantly greater than that of the NE0 surface (*p* < 0.05) (Fig. 7). In addition, the ALP activity on all the patterned surfaces was higher than that of the AM0 surface (*p* < 0.05). The ALP activity of the cells was increased with increasing width of the grooves and ridges. ALP activities of the cells on the AM90, 120, 150 surfaces were significantly greater than that for the AM30 and 60 surface (*p* < 0.05). AM180 showed the highest ALP activity (*p* < 0.05). The calcium concentration on the AM0 surface after 21 days of osteogenic culture was significantly greater than that of the NE0 surface (*p* < 0.05) (Fig. 8). The calcium concentration on all of the patterned surfaces was higher than that of the AM0 surface (*p* < 0.05). The calcium concentration of the cells on the AM120 and AM150 surfaces was significantly greater than that for the AM30, AM60 and AM90 surfaces (*p* < 0.05). The highest calcium concentration on the other patterned surfaces was on the AM180 surface (*p* < 0.05). Cell activities and differentiation on microgroove alumina were increased by more than twice compared to a non-microgrooved alumina surface. Moreover, related to osteoblast differentiation, the expression of ALP, RUNX2, OSX, OCN and OPN were all significantly increased on the alumina microgroove surface. Figure 9 shows the gene expressions of hMSCs after 14 days of cell culture. The effect of microgrooves on the levels of expression of core genes related to the osteoblast differentiation of MSCs, the AM180 surface was found to upregulate the expression of ALP, RUNX2, OSX, OCN and OPN genes compared to cells grown on the AM0 and the other patterned surfaces. The expression of OSX and OPN was significantly different for all patterned surfaces and was increased by 2.5-fold on AM180 compared to AM0. The expression of ALP, RUNX2, OCN on the AM180 surface was increased by 1.5 ~ 2.0-folds compared to AM0. Cell adhesion in aligned cells is enhanced compared with spherically shaped cells [26]. According to A. Kappor et al., cells on non-microgroove surfaces are aligned randomly, while cells adjacent to microgroove walls are aligned with the direction of the microgrooves, whereas cells positioned at the bottom of a microgroove are significantly less aligned [27]. Virginie Dumas et al. reported that various topographies can have an impact on altering cell morphology and controlling cell differentiation [28]. Therefore, it can be predicted that differences in cell morphology and cell shape on the AM0 and patterned surfaces will affect the degree of cell response. In other studies, the groove width and depth size have been reported to significantly influence cell shape and growth [29–31]. Most studies suggest that cells on narrow, 10 μm grooves were more elongated than cells that were grown on wide grooves [8]. Early works that were focused on cell alignment, demonstrated that microgrooves with narrow widths confer string cell guidance [1, 29, 32].

In this study, an alumina surface was found to contain more hydroxyl groups, positive charge and cell response than a titanium surface. These results indicate that the surface chemistry and charge of a material influence the response of MSCs. Microgrooves on an alumina surface were also found to decrease WCA in the parallel direction and enhanced the cell response. Mass transport could occur effectively in the parallel direction of a trench structure. From this study, it can be concluded that cell activity, i.e., differentiation and mineralization could be enhanced by increasing the wettability and transportability on wider microgrooves of a micro-molded alumina substrate. For confirmation of these results, further evaluations will be needed to identify the specific factors responsible and to optimize the width of grooves on an alumina surface in a wider range.

## Conclusion

We evaluated the activity of human mensenchymal stem cell on titanium, non-microgrooved alumina and microgrooved alumina prepared by a micro-molding technique using a replica of a PDMS mold. The alumina surface showed superior biological responses of osteoblast cells compared to titanium. These results indicate that higher concentrations of hydroxyl groups and positive charges of the alumina surface enhance cell activity compared to a titanium surface. The main findings were that the variation in the surface topography of alumina by microgrooves has an important impact on the behavior of hMSCs including the adhesion, differentiation of osteoblasts and osteoblast-specific gene expression. The significant increase in hMSC activity can be explained by an increase in material transportation in the parallel direction by increasing the cross-sectional area of a trench and by the extending the spreading distance in the perpendicular direction. The result is that the interruption against the groove walls in microgrooves having a wider width than 90 μm is decreased. Our results demonstrate that altering the topography of alumina by a micromolding process enhanced the wettability and isotropicity of the water droplet, which are important factors that influence the transport and propagation of materials on an alumina surface, therefore the cell activity of hMSCs including adhesion, differentiation of osteoblasts and osteoblast-specific gene expression are enhanced.
